# 电视纵隔镜在PET肺癌纵隔淋巴结显像阳性病例中的应用

**DOI:** 10.3779/j.issn.1009-3419.2010.02.18

**Published:** 2010-02-20

**Authors:** 宝东 刘, 修益 支, 庆生 许, 毅 张, 雷 苏, 东红 陈, 若天 王, 牧 胡, 磊 刘, 坤 钱

**Affiliations:** 100053 北京，首都医科大学肺癌诊疗中心，首都医科大学宣武医院胸外科 Department of Thoracic Surgery, Xuanwu Hospital, Lung Cancer Certer, Capital Medical University, Beijing 100053, China

**Keywords:** 肺肿瘤, 分期, 纵隔镜, 正电子发射断层成像, Lung neoplasms, Neoplasm staging, Mediastinoscopy, Positron emission tomography

## Abstract

**背景与目的:**

正电子发射断层成像检查（positron emission tomography, PET）作为非小细胞肺癌无创分期的手段有逐年增加的趋势，但是它在纵隔淋巴结分期中的作用尚不明了。本文探讨了电视纵隔镜检查术在PET肺癌纵隔淋巴结显像阳性病例中的临床价值。

**方法:**

2003年11月-2008年11月，对宣武医院收治的术前PET检查提示纵隔淋巴结转移的肺癌患者行电视纵隔镜检查术。对纵隔淋巴结进行病理学检查，病理来自纵隔镜或开胸清扫的纵隔淋巴结，分析纵隔镜诊断纵隔淋巴结转移的敏感性、特异性等。

**结果:**

本组61例肺癌患者中，男38例，女23例，平均年龄60岁（年龄41岁-81岁）。其中右肺癌41例，左肺癌20例。45例肺癌患者手术病理证实有纵隔淋巴结转移，其中10例N3的患者接受化疗，38例N2的患者给予2个周期的新辅助化疗，并根据检查结果确定是否接受开胸手术。16例无纵隔淋巴结转移的患者翻身行开胸探查、肺癌切除、纵隔淋巴结清扫术。PET的阳性预测值为73.8%（45/61）。电视纵隔镜在肺癌纵隔淋巴结分期中的敏感性、特异性、准确性、阳性预测值和阴性预测值分别为93.8%（45/48）、100%（13/13）、95.1%（58/61）、100%（45/45）、81.3%（13/16）。

**结论:**

PET在肺癌纵隔淋巴结分期中的阳性预测值较低，电视纵隔镜仍然是肺癌纵隔淋巴结分期的金标准。

## Abstract (en)

**Background and objective:**

Positron emission tomography (PET) is used increasingly in staging of non-small cell lung cancer (NSCLC) as a non-invasive tool. However, the role of PET in mediastinal lymphatic staging of NSCLC is not clear. The aim of this study was to demonstrate the efcacy of mediastinoscopy in determining mediastinal lymphatic metastasis in cases of positive PET fnding.

**Methods:**

We performed PET preoperatively in 68 patients with clinically operable NSCLC between 2003 and 2008. Mediastinal lymphatic defned as metastasis by PET (SUVmax>2.5) was recorded. Mediastinoscopy being performed initially in all patients. Involvement of mediastinal lymph nodes was verifed to compare the sensitivity and specifcity of mediastinoscopy and the related PET results.

**Results:**

From 2003 to 2008, 61 mediastinoscopy were performed. There were 38 men and 23 women, aged from 41 to 81 years (mean 60 years). Localization of the tumor was right lung in 41 patients and lef lung in 20 patients. Afer the operation, 45 patients were demonstrated to have N2 or N3 disease. Then patients with N3 mediastinal metastasis for chemotherapy, 38 patients with N2 mediastinal metastasis for neuadiuvant chemotherapy while lung resection and systemic mediastinal lymphatic dissection through thoracotomy was performed in the remaining 16 patients with no mediastinal metastasis. The positive prediction value of PET scan was 73.8% (45/61). The sensitivity, specifcity, accuracy, positive prediction value and negative prediction value in diagnosis of metastasis of mediastinal lymph nodes were 93.8% (45/48), 100% (13/13), 95.1% (58/61), 100% (45/45), 81.3% (13/16) for mediastinoscopy, respectively.

**Conclusion:**

PET results do not provide acceptable accuracy rates. Mediastinoscopy still remains the gold standard for mediastinal staging of NSCLC.

非小细胞肺癌（non-small cell lung cancer, NSCLC）治疗前分期对确定治疗方案和判断预后十分重要。特别是纵隔淋巴结转移十分活跃，上叶癌存在下纵隔淋巴结转移，下叶癌存在上纵隔淋巴结转移，还可能存在跳跃式淋巴结转移，因此在临床工作中，影像学诊断的纵隔淋巴结转移者，应通过纵隔淋巴结活检证实。纵隔镜检查是评价肺癌纵隔淋巴结转移分期的金标准，准确性高。然而，此项检查是一项有创操作，需要在全麻下进行，有一定的并发症；随着PET等先进诊断技术在临床上的应用，目前有逐渐替代纵隔镜的趋势。本文研究了电视纵隔镜在PET肺癌纵隔淋巴结显像阳性病例中的临床价值。

## 1 资料与方法

### 1.1 一般资料

2003年11月-2008年11月，本组61例肺癌患者，男38例，女23例，平均60岁（年龄41岁-81岁）。右肺癌41例，左肺癌20例。术前包括胸部X线平片、胸部螺旋CT、纤维支气管镜、痰脱落细胞学、肿瘤标志物等常规检查证实为肺癌。所有患者行PET检查提示纵隔淋巴结转移且无远处转移，根据PET淋巴结转移结果常规行电视纵隔镜检查。对严重贫血或凝血机制不全、胸主动脉瘤特别是主动脉弓的动脉瘤、严重的上腔静脉综合征、严重的心肺功能不全、严重的颈关节炎、颈椎强直不能后仰者、气管切开造口者视为电视纵隔镜手术禁忌证。

### 1.2 方法

#### 1.2.1 电视纵隔镜检查

61例患者中，颈部电视纵隔镜检查59例（右肺癌41例，左肺癌18例），主要获取2R、4R和7组淋巴结。胸骨旁电视纵隔镜检查2例（均为左上肺癌），手术探查第5组淋巴结，特别适用于左上叶肺癌。

#### 1.2.2 统计学处理

对电视纵隔镜或开胸纵隔淋巴结清扫的纵隔淋巴结常规进行石蜡切片，HE染色。以病理诊断为金标准，分析电视纵隔镜的敏感性、特异性、准确性、阳性预测值（1-假阳性）和阴性预测值（1-假阴性）。

## 2 结果

61例电视纵隔镜淋巴结活检的患者中，4例活检2R组淋巴结，35例活检4R组淋巴结，14例活检2R、4R组淋巴结，4例活检4R、7R组淋巴结，2例活检2R、4R、7R组淋巴结，2例活检第5组淋巴结。45例肺癌患者电视纵隔镜淋巴结活检病理证实有纵隔淋巴结转移，其中N2 35例（右肺癌28例，左肺癌7例）、N3 10例阳性（均为左肺癌），病理分型为腺癌19例、鳞癌17例、大细胞癌1例、未分型癌细胞8例。N2纵隔淋巴结转移根据能否清扫彻底决定是否开胸手术；N3患者放弃手术，接受放、化疗。16例无纵隔淋巴结转移的肺癌患者接受开胸肺癌切除、纵隔淋巴结清扫术，术后病理证实无纵隔淋巴结转移13例（右肺癌10例，左肺癌3例），有纵隔淋巴结转移3例（均为右肺癌，2例腺癌，1例鳞癌）。PET检查纵隔淋巴结的阳性预测值为73.8%（45/61）。电视纵隔镜在肺癌纵隔淋巴结分期中的敏感性、特异性、准确性、阳性预测值和阴性预测值分别为93.8%（45/48）、100%（13/13）、95.1%（58/61）、100%（45/45）、81.3%（13/16）。

本组中电视纵隔镜检查无手术死亡，2例术中出血，经过纱布压迫以后止血；2例术中发现右侧胸膜破裂有气泡逸出，局部填塞明胶海绵，术后未发现气胸；1例术中气管损伤，开胸修补；1例有切口血肿保守治疗后好转。

## 3 讨论

NSCLC术前临床TNM分期和术后病理TNM分期的一致性仅为45%，最主要的影响因素是对纵隔淋巴结（N）状态的判断。N3的NSCLC（Ⅲb期）患者不是手术适应证，但是对N2的NSCLC（Ⅲa期）患者的治疗选择至今仍有争议，我国的情况不同于西方国家，一直以来大部分的N2是外科的治疗对象。美国胸科医生学院同意对N2的NSCLC（Ⅲa期）进一步细分为Ⅲa1、Ⅲa2、ⅢAa3、Ⅲa4，并在2007年进一步修订并达成共识^[1]^。对N2（Ⅲa1-2）患者可以进行肺切除和纵隔淋巴结清扫，体力状况（PS）评分良好的患者，推荐含铂两药方案的术后辅助化疗。而对N2（Ⅲa3）的NSCLC患者，国外近年来的研究结果证实新辅助化疗能够控制并缩小病灶、降期，减少术中肿瘤种植，提高根治性手术切除率，尤其是化疗有效患者的手术完全切除率有明显提高，但对提高生存率的作用并不明显。而对N2（Ⅲa4）、PS评分良好的NSCLC患者可以进行放化疗。因此术前对N状态的评价十分重要。

术前纵隔淋巴结分期分无创和有创两种方法。在无创方法中，CT和PET是两个最主要的手段，CT目前仅作为筛选手段，而PET对NSCLC纵隔淋巴结分期的敏感性、特异性及准确率均高于CT^[2]^。从1996年-2006年的14个研究中，可评估的NSCLC患者2 865例，PET的敏感性为74%（95%CI: 69%-79%），特异性为85%（95%CI:82%-88%），但同样存在假阳性的问题^[3]^。因此，影像学诊断为N2的患者，需通过纵隔淋巴结病理检查以进一步确诊^[4]^。在有创检查中，经支气管镜针吸活检（transbronchial needle aspiration, TBNA）、超声引导下经支气管镜针吸活检（ultrasound-guided bronchoscopy with fine needle aspiration, EBUS-FNA）和食管内镜超声引导下针吸活检（endoscopic esophageal ultrasound-guided fine needle aspiration, EUS-FNA）的阴性预测值低，目前开展并不广泛^[5, 6]^，纵隔镜仍然是NSCLC纵隔淋巴结分期的最常用手段。

有学者^[7-9]^比较了PET和纵隔镜在NSCLC术前N分期后认为，PET检查纵隔淋巴结阴性的患者可不必行纵隔镜手术，提示PET具有很高的阴性预测值。本组中PET检查纵隔淋巴结阳性的NSCLC患者中，电视纵隔镜手术在术前纵隔淋巴结分期中的敏感性和特异性可分别达到90%以上和100%，而PET检查的假阳性率达26.2%，即大约每4个PET检查可疑纵隔淋巴结转移的病人有1例为假阳性，可能与我们的病人高龄、合并肺部感染等有关。因此PET检查纵隔淋巴结阳性的患者仍然需要进行纵隔镜手术，与2008年版美国版NCCN NSCLC临床指引一致。本组中电视纵隔镜手术假阴性率达18.75%（3/16），有文献认为其中一半的原因是手术探查没有到达转移淋巴结的部位而出现漏诊^[3]^。但是经颈纵隔镜发现N2阳性患者的预后与N2阴性者明显不同，即使纵隔镜检查N2阴性的患者，开胸手术以后证实N2为阳性，预后也完全不同。

与传统纵隔镜手术相比，电视纵隔镜手术有以下优点^[10]^：能够提供清晰放大的视野，提高术者对精细解剖结构的辨别，提高了这一技术的安全性和准确性；在助手的有效配合下，术者得以双手进行操作，大大提高术中操作的灵活性以及活检的准确性，明显缩短手术时间。

随着手术经验的积累，电视纵隔镜的手术适应证也在不断扩大，如本组对2例合并上腔静脉综合征的肺癌进行了经颈电视纵隔镜淋巴结活检，没有出现大出血^[11]^。

本组发生2例术中出血，出血最常见的是供应纵隔淋巴结的支气管动脉分支出血，特别是供应隆突下淋巴结的分支，淋巴结创面及滋养血管出血可通过电凝或明胶海绵或止血纱布填塞、纱布压迫止血。2例术中可疑胸膜破裂，可见气泡冒出，发生在右侧。常由钝性分离动作过大过猛、活检时误伤胸膜而引起。不要急于手术修补，可用明胶海绵或止血纱布填塞损伤处，缝合切口。气管损伤1例，多发生于初学者，肿瘤与气管致密粘连或侵犯气管时容易发生，一旦发生，必须经胸骨正中切开加以修补。本组颈部纵隔镜切口血肿1例，经过保守治疗后好转。这些并发症发生机率较少且不致命，因此电视纵隔镜作为肺癌纵隔淋巴结分期的金标准将会得到广泛的应用。
